# Plant epithelia: What is the role of the mortar in the wall?

**DOI:** 10.1371/journal.pbio.3000073

**Published:** 2018-12-05

**Authors:** Michael Palmgren

**Affiliations:** Department of Plant and Environmental Sciences, University of Copenhagen, Copenhagen, Denmark

## Abstract

In a growing plant root, the inner vascular system is sealed off by an epithelium, the endodermis. The space between all of the cells in the endodermal layer is filled with an impermeable mass called the Casparian strip, which closes the spaces between cells in the endodermal layer. The role of the Casparian strip has been proposed to prevent backflow of water and nutrients into the soil, but as mutant plants lacking the Casparian strip only have weak phenotypes, the view that it serves an essential function in plants has been challenged. In an accompanying paper, it is shown that loss of the Casparian strip impacts the ability of the plant to take up ammonium and allocate it to the shoots.

## Abstract 2

What is the function of the Casparian strip, a plant structure first described in 1865? This Primer explores the implications of a new study which reports that loss of the Casparian strip in root endodermis affects the ability of the plant to take up ammonium and allocate it to the shoots.

Anyone who has carefully chewed a carrot knows that, when crushed, it is possible to separate it into an outer layer and an inner sweet-tasting rod. This rod includes the vascular cylinder of the root. Whereas a young growing root as such is covered by an epithelium, the epidermis, the vascular cylinder, with its vasculature, is covered by yet another epithelium, the endodermis (Fig 1A). All space between the cells in this layer is filled with a water-impermeable mass that effectively separates the extracellular space in the vascular cylinder from that of the outer layer [1]. This filling, like mortar in a brick wall but made of lignin [2], is called the Casparian strip, named after the German botanist Robert Caspary, who first described it in 1865 (Fig 1B and Fig 2) [3].

**Fig 1:**
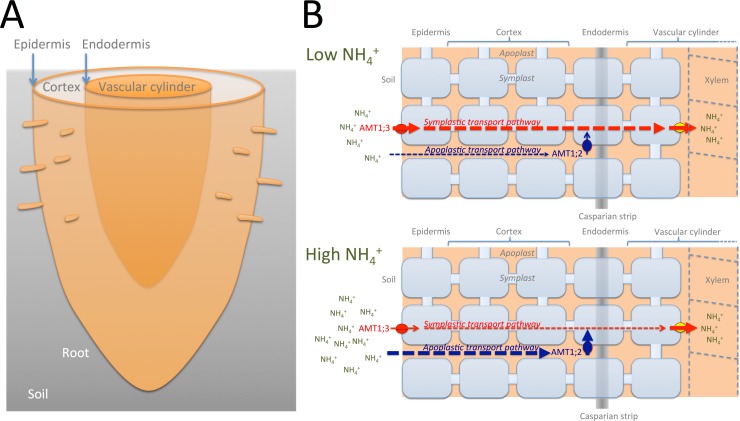
Apoplastic and symplastic transport pathways of ammonium into the root vascular cylinder. A. The 3D tissue layout of a root is a series of concentric annular cylinders made up of the epidermis, cortex, endodermis, and, in the center, the vascular cylinder [22]. B. The Casparian strip is a water-impermeable sealing that fills the space between cells of the root endodermis. The only way for water and solutes to pass this barrier is to enter an endodermal cell. This can happen in one of two ways. Either water or solutes are taken up by transporters at the root epidermis (here, in the case of ammonium, by AMT1;3) and move from cell to cell in a symplastic continuum into an endodermal cell. This symplastic transport pathway predominates when external ammonium is low. Alternatively, water and solutes from the outside medium move through cell walls toward the endodermis and, at this point, are taken up by a transporter in this cell layer (here, in the case of ammonium, by AMT1;2). This apoplastic transport pathway predominates when external ammonium is high. Some solutes can enter the symplast from the apoplast via any cell in the outer root: the epidermal cells, the cortex cells, or the endodermal cells. In both cases, water and solutes pass the Casparian strip by diffusing through an endodermal cell and are subsequently loaded into the xylem by other transporters for long-distance transport to the shoot. AMT, ammonium transporter.

**Fig 2:**
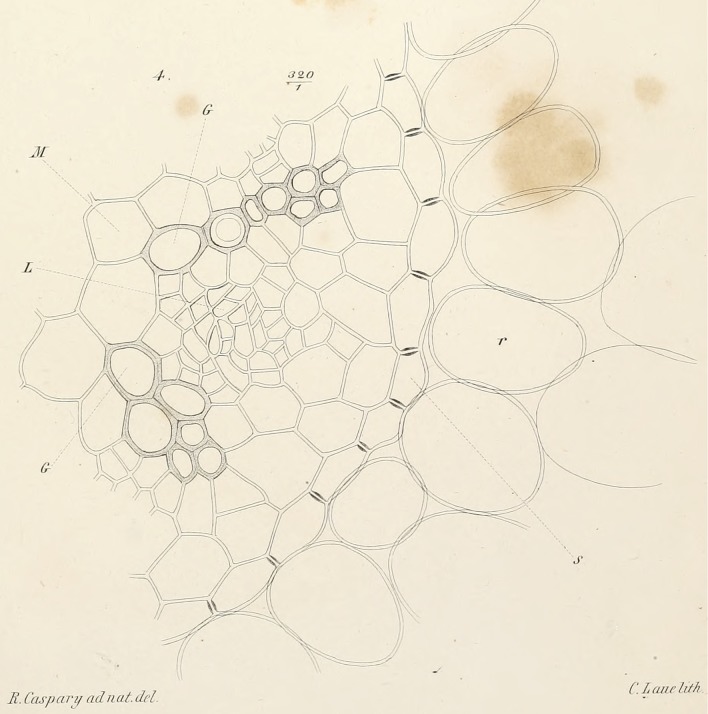
Cross-section of root of *Ficaria verna* Huds. From Robert Caspary’s original drawing of the Casparian strip from Fig 4 in reference [3]. The Casparian strip is seen as a black thickening between endodermal cells. Magnification x 320. G, “Gefässgruppen” (vessel groups; xylem); L, “Leitzellen” (conducting cells; phloem); M, “Mark” (pith); r, “Rindenzellen” (cortical cells); S, “Schutzscheide” (protective sheath; endodermis).

Since the Casparian strip is resistant to treatment with even concentrated sulfuric acid [3,4], which readily degrades all other cell wall constituents, it was suggested that the Casparian strip serves as an impregnating layer that prevents water and solutes from diffusing into the vascular cylinder and also prevents back leakage of solution under pressure from it [5,6]. That the Casparian strip indeed is an impermeable belt was demonstrated by exposing roots to uranyl [7] and lanthanum [8]. These tracer ions diffuse in from the medium in the space between cells. Electron microscopy revealed that both ions form electron-dense deposits on cells from the epidermis all the way to the endodermis but not beyond this cell layer.

The vascular cylinder contains two separate vascular systems, the xylem and the phloem. We humans also have two types of vascular systems, arteries and veins, but they both transport the same thing, namely blood. In plants, each of the two vascular systems transport specific solutes and typically in different directions. The xylem delivers mineral nutrients taken up from the soil to the shoot, and the phloem transports sugars and other products of photosynthesis from the leaves to the root and other places of the plant in need of energy. In contrast to the phloem (and arteries and veins), the xylem is made of dead cells with porous cell walls; the vessels are therefore not isolated from their surroundings and their content is free to leak out of them. This explains why, in the absence of an endodermal barrier, pressure would never be able to build up in the vascular cylinder.

If nutrients in the xylem cannot leak out of the vascular cylinder, how are they able enter it initially? Entrance into the vascular cylinder is made possible by the millions of cells of the endodermis that are embedded in the Casparian strip like bricks in a wall. In order to enter the vascular cylinder, a nutrient must actively enter an endodermal cell through a transport protein, diffuse through it, and in doing so, bypass the Casparian strip (Fig 1B and Fig 2). The whole process is like a car driving through a tunnel in a hill. On the other side of the Casparian strip, the nutrient is transported by another transport protein out of the cell, transported into the xylem, and is then free to move upward toward the shoot. However, this function in permeating nutrients only holds on as long as endodermal cells are young. With progressing maturation, endodermal cells become impregnated by a layer of waxy substances that are deposited at the inner surface of the cell walls. This so-called suberization prohibits nutrients to enter or leak out, thereby sealing the endodermis like the peel of an orange [9,10].

A nutrient in the soil can be transported through an endodermal cell via two transport pathways (Fig 1B): i) either it is first taken up by transport proteins in the epidermal cells at the root–soil interface from which it diffuses from cell to cell through plasmadesmata, structures that, analogous to gap junctions in animal cells, form bridges between cells and ultimately find their way to endodermal cells; or ii) it diffuses all the way from the soil through the highly permeable cell walls between cells to the Casparian sealing, and during this passage, it is at some point actively taken up by cells, ultimately endodermal cells. The first pathway is called the symplastic pathway because the nutrient moves inside cells in a symplastic continuum; the other is the apoplastic pathway, as the main part of the diffusion occurs outside of cells.

The signaling cascade that triggers the formation of Casparian strips starts with small peptides derived from the vascular cylinder [11,12]. Wherever they leak out of the vascular cylinder, they are recognized by SCHENGEN3 (SGN3), a receptor-like kinase located in the plasma membrane at the distal side of endodermal cells [13]. This sparks an intricate developmental program, in which the transcription factor Myb-related protein 36 (MYB36) plays a central role in the synthesis and deposition of lignin between endodermal cells [14,15]. The function of the Casparian strip becomes more important under stress conditions such as high salt [16]. However, under salt stress, the Casparian strip itself is relatively unmodified, whereas the suberin layer expands dramatically [17]. This shows that the deposition of suberin may be essential to avoid passage of Na^+^ and Cl^−^ through endodermal cells.

The *schengen3* mutant of the model plant *Arabidopsis thaliana* is unable to form a functional Casparian strip and, in contrast to other Casparian strip mutants, possesses no compensatory mechanism to seal off the endodermis [13]. This mutant offers a unique system to understand the role of the endodermal barrier in plants. Surprisingly, studies of this mutant have shown that SGN3 is not essential for plant life. In fact, the phenotype of *sgn3* plants is very weak in the sense that the mutant plants take up water and nutrients and apparently grow normally. An exception is the macronutrient potassium, which, in mutant shoots, is lower than in the wild type [13]. This led to the suggestion that the Casparian strip might not be essential for water and nutrient uptake as such but could have more specific roles, such as permitting accumulation of potassium.

A study published today in *PLOS Biology* has investigated whether loss of *SGN3* affects transport from roots to shoots of another important nutrient, namely ammonium [18]. First, it is shown that strontium, an element not required by plants and typically transported along the apoplastic pathway, does not enter the vasculature in wild-type plants but readily does so in the *sgn3* mutant plants. This confirms the role of the Casparian strip as a barrier for solute flow from the growth medium into the vascular cylinder. However, no matter whether the Casparian strip was present or not, ammonium was transported from the root to the shoot. There are different transport proteins in roots involved in ammonium uptake and xylem loading [19,20]. By genetically crossing of *sgn3* with other lines expressing ammonium transporters only in the epidermis or in the endodermis, the contribution of each transporter in the presence and absence of the Casparian strip could be studied. With a functional Casparian strip and when soil ammonium content is low, uptake at the epidermis followed by symplastic transport was shown to prevail. In contrast, when soil ammonium content is high, transport via the apoplastic route followed by uptake at the endodermis became the dominant route. In the absence of the Casparian strip, the efficiency of the apoplastic transport route decreased. However, unexpectedly, the efficiency of the symplastic pathway increased in the absence of the Casparian strip, which implies that the endodermal bypass caused by the *sgn3* mutation has opposing consequences for the symplastic and apoplastic transport pathways. This was a surprising result, as it indicates that the apoplastic route requires Casparian strips to render ammonium uptake at the endodermis more efficient. Moreover, this approach allowed for the first time determining the relative contribution of both pathways and their dependencies on the Casparian strip for any nutrient.

A root pressure develops when mineral nutrients get actively loaded into the xylem and are followed by water. The *sgn3* mutant has reduced root pressure and slower flow of water through the xylem most likely because water cannot build up in this structure without endodermal sealing [13]. Water evaporating from the leaves of the shoots creates a low pressure in the shoot xylem that allows its contents to move upward. Under conditions of high transpiration and accompanying suction of xylem sap from the root, nutrient backflow is prevented, and the Casparian strip may therefore not be as important as when transpiration is low. In an earlier study, sunflower plants growing in water baths were fed with nutrients during the day or the night, when transpiration is high and low, respectively [21]. When comparing the plants under the two conditions, no differences in nutrient uptake and growth could be observed. It was thus concluded that convective water transport in the xylem, brought about by root pressure, is in itself sufficient to supply the shoot with nutrients taken up from the soil [21].

Strikingly, in the new study, plants were grown under conditions—liquid medium and strong light—for which transpiration is high and root pressure low. This could explain the weak nutrient-related phenotypes of the mutant plants. It will be interesting in future work to see how mutant plants without a functional Casparian strip behave under conditions for which the root pressure is high and transpiration low.

## Abbreviations

MYB36: Myb-related protein 36
SGN3: SCHENGEN3

## References

[1] Geldner N. The endodermis. Annu Rev Plant Biol. 2013;64: 531–558 10.1146/annurev-arplant-050312-120050 23451777 10.1146/annurev-arplant-050312-120050

[2] Naseer S, Lee Y, Lapierre C, Franke R, Nawrath C, Geldner N. Casparian strip diffusion barrier in Arabidopsis is made of a lignin polymer without suberin. Proc Natl Acad Sci U S A. 2012;109: 10101–10106. 10.1073/pnas.1205726109 22665765 10.1073/pnas.1205726109 PMC3382560

[3] Caspary R. Bemerkungen über die Schutzscheide und die Bildung des Stammes und der Wurzel. Jahrbücher für wissenschaftliche Botanik 1865;4: 101–124.

[4] Krömer K. Wurzelhaut, Hypodermis und Endodermis der Angiospermenwurzel. Bibl. Bot. 1903;43: 1–59.

[5] Priestley JH, North EE. Physiological studies in plant anatomy: III. The structure of the endodermis in relation to its function. New Phytol. 1922;3: 113–139.

[6] Crafts AS, Broyer TC. Migration of salts and water into xylem of the roots of higher plants. Am. J. Bot. 1938;25: 529–535.

[7] Robards AW, Robb ME. Uptake and binding of uranyl ions by barley roots. Science. 1972;178: 980–982. 10.1126/science.178.4064.980 17774510 10.1126/science.178.4064.980

[8] Nagahashi G, Thomson WW, Leonard RT. The casparian strip as a barrier to the movement of lanthanum in corn roots. Science 1974;183: 670–671. 10.1126/science.183.4125.670 17778842 10.1126/science.183.4125.670

[9] Kolattukudy PE. Biopolyester membranes of plants: cutin and suberin. Science 1980;208: 990–1000 10.1126/science.208.4447.990 17779010 10.1126/science.208.4447.990

[10] Doblas VG, Geldner N, Barberon M. The endodermis, a tightly controlled barrier for nutrients. Curr Opin Plant Biol. 2017;39: 136–143. 10.1016/j.pbi.2017.06.010 28750257 10.1016/j.pbi.2017.06.010

[11] Doblas VG, Smakowska-Luzan E, Fujita S, Alassimone J, Barberon M, Madalinski M, et al Root diffusion barrier control by a vasculature-derived peptide binding to the SGN3 receptor. Science 2017;355: 280–284. 10.1126/science.aaj1562 28104888 10.1126/science.aaj1562

[12] Nakayama T, Shinohara H, Tanaka M, Baba K, Ogawa-Ohnishi M, Matsubayashi Y. A peptide hormone required for Casparian strip diffusion barrier formation in Arabidopsis roots. Science 2017;355: 284–286. 10.1126/science.aai9057 28104889 10.1126/science.aai9057

[13] Pfister A, Barberon M, Alassimone J, Kalmbach L, Lee Y, Vermeer JE et al A receptor-like kinase mutant with absent endodermal diffusion barrier displays selective nutrient homeostasis defects. Elife 2014;3: e03115 10.7554/eLife.03115 25233277 10.7554/eLife.03115 PMC4164916

[14] Kamiya T, Borghi M, Wang P, Danku JM, Kalmbach L, Hosmani PS et al The MYB36 transcription factor orchestrates Casparian strip formation. Proc Natl Acad Sci U S A. 2015;112: 10533–10538. 10.1073/pnas.1507691112 26124109 10.1073/pnas.1507691112 PMC4547244

[15] Li P, Yu Q, Gu X, Xu C. Qi S, Wang H et al Construction of a functional Casparian strip in non-endodermal lineages is orchestrated by two parallel signaling systems in Arabidopsis thaliana. Curr Biol. 2018;28: 2777–2786 10.1016/j.cub.2018.07.028 30057307 10.1016/j.cub.2018.07.028

[16] Karahara I, Ikeda A, Kondo T, Uetake Y. Development of the Casparian strip in primary roots of maize under salt stress. Planta 2004;219: 41–47. 10.1007/s00425-004-1208-7 14986139 10.1007/s00425-004-1208-7

[17] Barberon M, Vermeer JE, De Bellis D, Wang P, Naseer S, Andersen TG et al,Adaptation of root function by nutrient-induced plasticity of endodermal differentiation. Cell 2016;164: 447–459. 10.1016/j.cell.2015.12.021 26777403 10.1016/j.cell.2015.12.021

[18] Duan F, Giehl RFH, Geldner N, Salt DE, von Wirén N. Root zone-specific localization of AMTs determines ammonium transport pathways and nitrogen allocation to shoots. PLoS Biol. 2018; (in press, this issue) 10.1371/journal.pbio.2006024 PMC6218093 30356235

[19] Yuan L, Loqué D, Kojima S, Rauch S, Ishiyama K, Inoue E et al The organization of high-affinity ammonium uptake in Arabidopsis roots depends on the spatial arrangement and biochemical properties of AMT1-type transporters. Plant Cell 2007;19: 2636–2652. 10.1105/tpc.107.052134 17693533 10.1105/tpc.107.052134 PMC2002620

[20] Giehl RFH, Laginha AM, Duan F, Rentsch D, Yuan L, von Wirén N. A critical role of AMT2;1 in root-to-shoot translocation of ammonium in Arabidopsis. Mol. Plant 2017;10: 1449–1460. 10.1016/j.molp.2017.10.001 29032248 10.1016/j.molp.2017.10.001

[21] Tanner W, Beevers H. Transpiration, a prerequisite for long-distance transport of minerals in plants? Proc Natl Acad Sci U S A 2001;98: 9443–9447. 10.1073/pnas.161279898 11481499 10.1073/pnas.161279898 PMC55440

[22] Foster KJ, Miklavcic SJ. A Comprehensive Biophysical Model of Ion and Water Transport in Plant Roots. I. Clarifying the Roles of Endodermal Barriers in the Salt Stress Response. Front Plant Sci. 2017;8: 1326 10.3389/fpls.2017.01326 28804493 10.3389/fpls.2017.01326 PMC5532442

